# Hypoxia regulates RhoA and Wnt/β-catenin signaling in a context-dependent way to control re-differentiation of chondrocytes

**DOI:** 10.1038/s41598-017-09505-6

**Published:** 2017-08-22

**Authors:** Ece Öztürk, Stefanie Hobiger, Evelin Despot-Slade, Michael Pichler, Marcy Zenobi-Wong

**Affiliations:** Cartilage Engineering+ Regeneration Laboratory, ETH Zurich, Otto-Stern-Weg 7, 8093 Zurich, Switzerland

## Abstract

Cartilage tissue is avascular and hypoxic which regulates chondrocyte phenotype via stabilization of HIFs. Here, we investigated the role of hypoxia and HIFs in regulation of Rho and canonical Wnt signaling in chondrocytes. Our data demonstrates that hypoxia controls the expression of RhoA in chondrocytes in a context-dependent manner on the culturing conditions. Within a 3D microenvironment, hypoxia suppresses RhoA on which hypoxia-driven expression of chondrogenic markers depends. Conversely, hypoxia leads to upregulation of RhoA in chondrocytes on 2D with a failure in re-expression of chondrogenic markers. Similarly to RhoA, hypoxic regulation of Wnt/β-catenin signaling depends on the microenvironment. Hypoxia downregulates β-catenin within 3D hydrogels whereas it causes a potent increase on 2D. Hypoxia-induced suppression of canonical Wnt signaling in 3D contributes to the promotion of chondrogenic phenotype as induction of Wnt signaling abrogates the hypoxic re-differentiation of chondrocytes. Inhibiting Wnt/β-catenin signaling via stabilization of Axin2 leads to a synergistic enhancement of hypoxia-induced expression of chondrogenic markers. The effects of hypoxia on Rho and Wnt/β-catenin signaling are HIF-dependent as stabilizing HIFs under normoxia revealed similar effects on chondrocytes. The study reveals important insights on hypoxic signaling of chondrocytes and how hypoxia regulates cellular mechanisms depending on the cellular microenvironment.

## Introduction

Articular cartilage tissue lacks vascularization and has limited access to the body’s healing mechanisms and therefore, a low capacity for regeneration once injured or degenerated. Current autologous cell-based cartilage repair strategies often involve *in vitro* monolayer expansion of chondrocytes to achieve sufficient number of cells to be implanted^1^. However, chondrocytes are not phenotypically stable on two-dimensional (2D) culture and lose the expression of cartilage-specific markers such as collagen 2 and aggrecan and adopt a fibroblastic phenotype with a concomitant upregulation of collagen 1^2^. Development of strategies to overcome such “de-differentiation” and induce re-expression of chondrogenic markers in order to ensure regeneration of biochemically and mechanically functional hyaline cartilage tissue still remains a challenge. Methods to biomimic cartilage tissue and provide chondrocytes with cues that are present in their native microenvironment include encapsulation in three-dimensional (3D) hydrogels, addition of growth factors from the TGF-β, FGF or BMP families and/or exposing the chondrocytes to native oxygen levels^3–5^.

Due to the avascular structure of cartilage tissue, chondrocytes reside in a hypoxic microenvironment with an oxygen content ranging from 5–10% O_2_ in the superficial zone to 1% O_2_ in the deep zone and adapt metabolically to chronic hypoxia^6–8^. Cultivation of cells under hypoxic conditions has been shown to stimulate re-differentiation of chondrocytes^9–12^ as well as regulate expression of zonal cartilage markers such as superficial zone protein^13^. Furthermore, hypoxia has been reported to promote both Sox-9-mediated chondrogenic differentiation of stem cells^14, 15^ and inhibit chondrocyte hypertrophy^16–18^. Cells respond to hypoxia through hypoxia-inducible factors (HIF), which constitute of a stable β subunit and an oxygen-controlled α subunit. Under normoxic conditions, the HIF-α subunit gets hydroxylated via prolyl hydroxylases (PHDs) which targets it to proteosomal degradation whereas under hypoxia, HIF-α is stabilized, complexes with its β subunit and drives the expression of its targets^19^. All the known HIFs (HIF-1α, 2α and 3α) are shown to be involved in the hypoxia-mediated regulation of chondrogenic phenotype. HIF-1α has been shown to be a critical survival factor for chondrocytes during development^20^, induce expression of cartilage markers in chondrocytes^21, 22^ and suppress expression of fibroblastic markers^23^. The role of HIF-2α, on the other hand, has been more controversial where it has been shown to induce cartilage matrix production and anabolism^24, 25^ as well as chondrocyte hypertrophy and catabolism^26^. Recently, HIF-3α has also been attributed a role in the hypoxic-regulation of cartilage markers^27^.

Besides the involvement and mediation of hypoxic re-differentiation by HIFs, other signaling mechanisms that are involved in the maintenance of chondrogenic phenotype under hypoxia remain largely unknown. Here, we investigated two signaling pathways known to negatively regulate chondrocyte phenotype, RhoA^28^ and canonical Wnt signaling^29^ pathways, in chondrocytes under hypoxic conditions. We hypothesized that hypoxia could suppress these signaling pathways in chondrocytes and we explored a possible role of the hypoxic regulation of these pathways in the induction of the re-expression of cartilage markers. This study aims at deepening the current knowledge on hypoxic signaling mechanisms in chondrocytes that would lead to improvement of cell-based cartilage repair strategies.

## Results

### Hypoxia promotes potent chondrocyte re-differentiation only within a 3D environment and in a HIF-dependent manner

**Figure 1:**
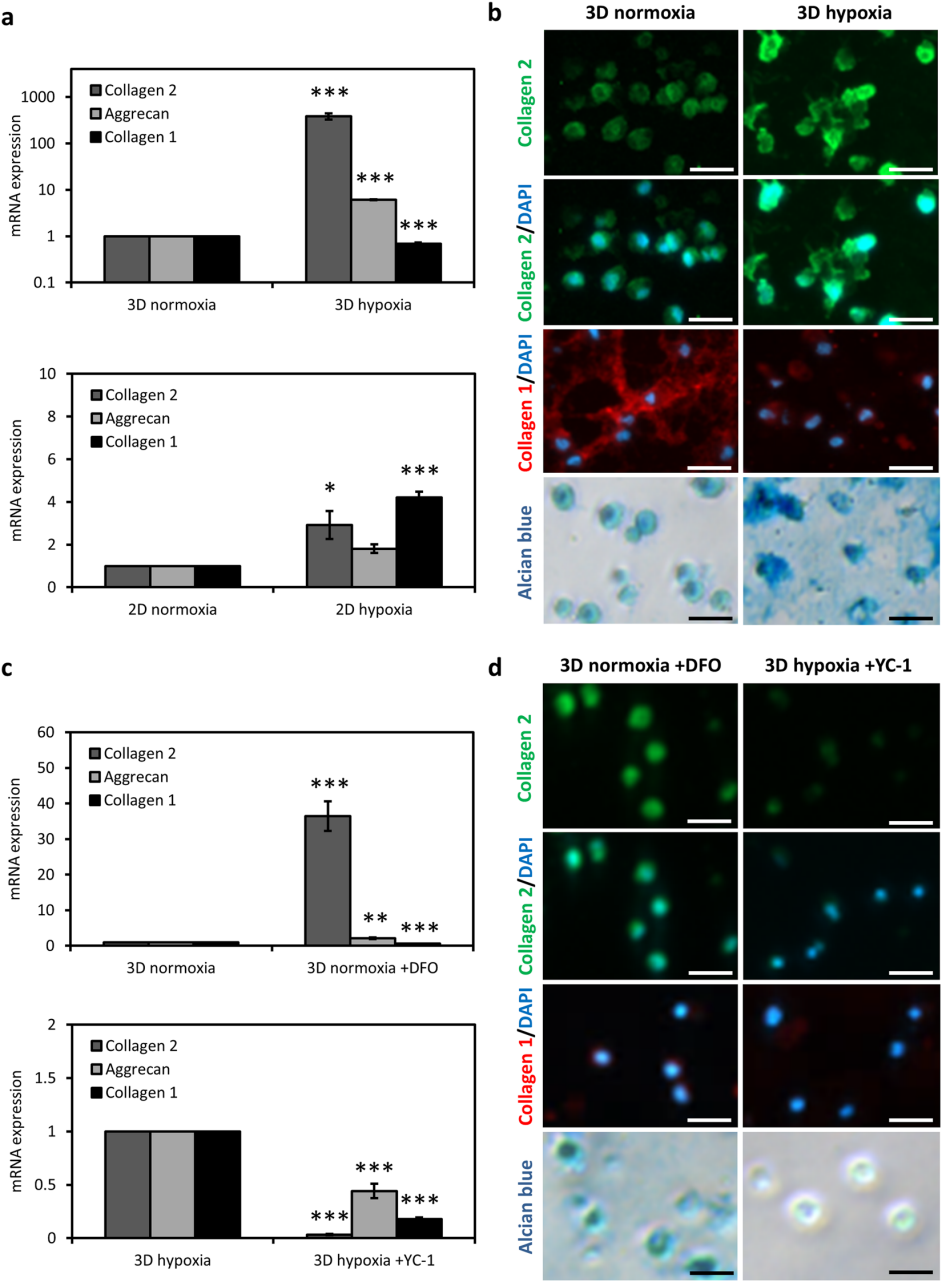
Hypoxia induces chondrogenic re-differentiation potently within a 3D context and in a HIF-dependent manner. (**a**) mRNA expression of collagen 2, collagen 1 and aggrecan of chondrocytes cultured in 3D alginate hydrogels or on 2D under normoxia (21% O_2_) or hypoxia (1% O_2_) for 7 days. Mean ± s.d. for n = 3; normalized to normoxia; *p < 0.05, ***p < 0.001 when compared to the normoxia sample. (**b**) Immunofluorescence imaging of collagen 2 (green) and collagen 1 (red) and alcian blue staining of chondrocytes in 3D alginate hydrogels under normoxia or hypoxia. DAPI (blue) was used for nuclei staining. Scale bar: 100 µm. (**c**) mRNA expression of collagen 2, collagen 1 and aggrecan of chondrocytes in 3D under normoxia with DFO (150 µM) treatment or under hypoxia with YC-1 (10 µM) treatment. Mean ± s.d. for n = 3; normalized to no treatment; **p < 0.01, ***p < 0.001 when compared to the no treatment sample. (**d**) Immunofluorescence imaging of collagen 2 (green) and collagen 1 (red) and alcian blue staining of chondrocytes in 3D under normoxia with DFO (150 µM) treatment or under hypoxia with YC-1 (10 µM) treatment. Scale bar: 25 µm.

Passaged chondrocytes were cultured either in alginate hydrogels (3D) or on tissue culture plastic (2D) under normoxic (21% O_2_) or hypoxic (1% O_2_) conditions for 7 days. Expression of markers of chondrocyte phenotype such as collagen 2 and aggrecan as well as the de-differentiation marker collagen 1 were assessed. In 3D hydrogels, collagen 2 (386.3 ± 61.1 fold) and aggrecan (6.1 ± 0.12 fold) gene expression showed a potent increase in response to hypoxia whereas collagen 1 expression showed a slight but significant decrease (Fig. 1a). On the other hand, when chondrocytes were cultured on 2D, the chondrogenic effect of hypoxia decreased drastically and only a minor increase in collagen 2 and aggrecan gene expression was observed. Furthermore, expression of collagen 1 mRNA increased significantly on 2D (Fig. 1a). Deposition of collagen 2 and collagen 1 was monitored by immunostaining and glycosaminoglycan (GAG) deposition was detected by alcian blue staining. In line with the gene expression data, collagen 2 deposition in chondrocytes was strongly enhanced by hypoxia in 3D hydrogels (Fig. 1b). Chondrocytes cultured under normoxic conditions were observed to deposit high amounts of collagen 1 which was suppressed under hypoxia. Alcian blue staining similarly demonstrated that hypoxic culturing conditions highly promoted GAG deposition in chondrocytes (Fig. 1b). On 2D, however, collagen 2 and collagen 1 deposition in chondrocytes cultured under normoxia and hypoxia did not show a big difference which correlated with the gene expression data (Figure S1). These results indicate that 3D culturing and hypoxic microenvironment exert a synergistic effect on the re-expression of cartilage-specific markers in passaged chondrocytes.

We next investigated the HIF-dependence of the chondrogenic effect of 3D culturing under hypoxia. For this, we treated chondrocytes with deferoxamine (DFO) to stabilize HIFs under normoxic conditions or with YC-1 to destabilize HIFs under hypoxic conditions. HIF-1α staining revealed protein stabilization only under hypoxia and YC-1 treatment led to the degradation of HIF-1α under hypoxic conditions (Figure S2). However, HIF-1α staining was unexpectedly only observed to be cytoplasmic under hypoxia which could be due to timing or a negative feedback mechanism on the transactivation of the protein (Figure S2). DFO treatment of chondrocytes in 3D caused a strong increase in collagen 2 expression (36.4 ± 4.17 fold) under normoxic conditions. Aggrecan expression similarly showed a significant increase (2.1 ± 0.26 fold) whereas expression of collagen 1 decreased (Fig. 1c). On the other hand, YC-1 treatment under hypoxia suppressed the chondrogenic effect of hypoxia on chondrocytes with a drastic loss of collagen 2 expression and a significant decrease in aggrecan expression. Interestingly, collagen 1 gene expression also showed a decrease in response to YC-1 treatment in chondrocytes (Fig. 1c). Immunofluorescence similarly showed an increase of collagen 2 deposition and a decrease in collagen 1 in DFO-treated chondrocytes in 3D under normoxia compared to non-treated controls (Fig. 1d). Under hypoxia, YC-1 treatment caused a potent loss of collagen 2 as well as GAG deposition of chondrocytes in 3D hydrogels (Fig. 1d). On 2D, HIF stabilization under normoxia with DFO treatment caused an increase in collagen 2 gene expression even further than hypoxia and collagen 1 expression was suppressed whereas HIF destabilization under hypoxia with YC-1 treatment caused a slight decrease in all markers on 2D (Figure S3).

### Hypoxia regulates RhoA differently in chondrocytes depending on the culturing conditions

**Figure 2:**
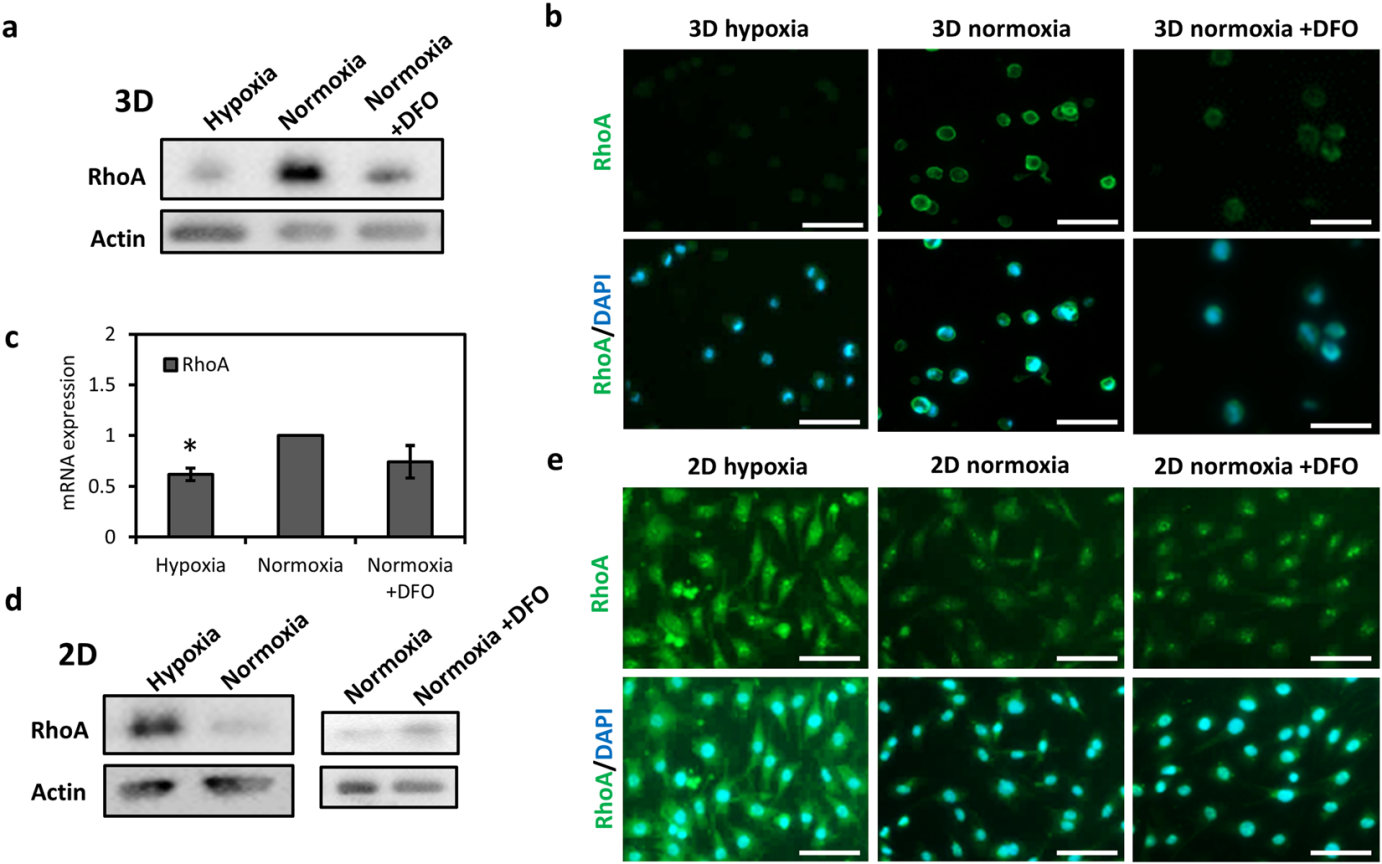
Hypoxia regulates RhoA differently depending on the culturing conditions in a HIF-mediated manner. (**a**) Western blot showing total RhoA protein in chondrocytes in 3D under hypoxia, normoxia and normoxia with DFO (150 µM) treatment. Actin was used as the loading control. (**b**) Immunofluorescence imaging of RhoA (green) and (**c**) mRNA expression of RhoA in chondrocytes in 3D under hypoxia, normoxia and normoxia with DFO (150 µM) treatment. Mean ± s.d. for n = 3; normalized to normoxia; *p < 0.05 when compared to normoxia. (**d**) Western blot showing total RhoA protein in chondrocytes on 2D under hypoxia, normoxia and normoxia with DFO (150 µM) treatment. Actin was used as the loading control. (**e**) Immunofluorescence imaging of RhoA (green) in chondrocytes on 2D under hypoxia, normoxia and normoxia with DFO (150 µM) treatment. Scale bar: 50 µm.

After observing a synergistic effect of hypoxia and 3D culturing on chondrocyte re-differentiation, we monitored the expression of cytoskeletal regulator RhoA in chondrocytes under the different oxygen conditions. In 3D hydrogels, hypoxia caused a suppression of RhoA protein in chondrocytes. This suppression was found to be HIF-dependent as similar downregulation of RhoA was observed under normoxia when the chondrocytes were treated with DFO (Fig. 2a,b). RhoA mRNA expression also slightly decreased in response to hypoxia or DFO treatment under normoxia in chondrocytes encapsulated in 3D hydrogels (Fig. 2c). Surprisingly, when the chondrocytes were cultured on 2D, hypoxia induced a strong increase in RhoA protein levels (Fig. 2d,e). Under normoxic conditions, HIF stabilization with DFO treatment caused a similar effect on RhoA protein however the increase was less pronounced than hypoxia (Fig. 2d).

### Activation of RhoA in 3D hinders the hypoxia-induced re-differentiation of chondrocytes

**Figure 3:**
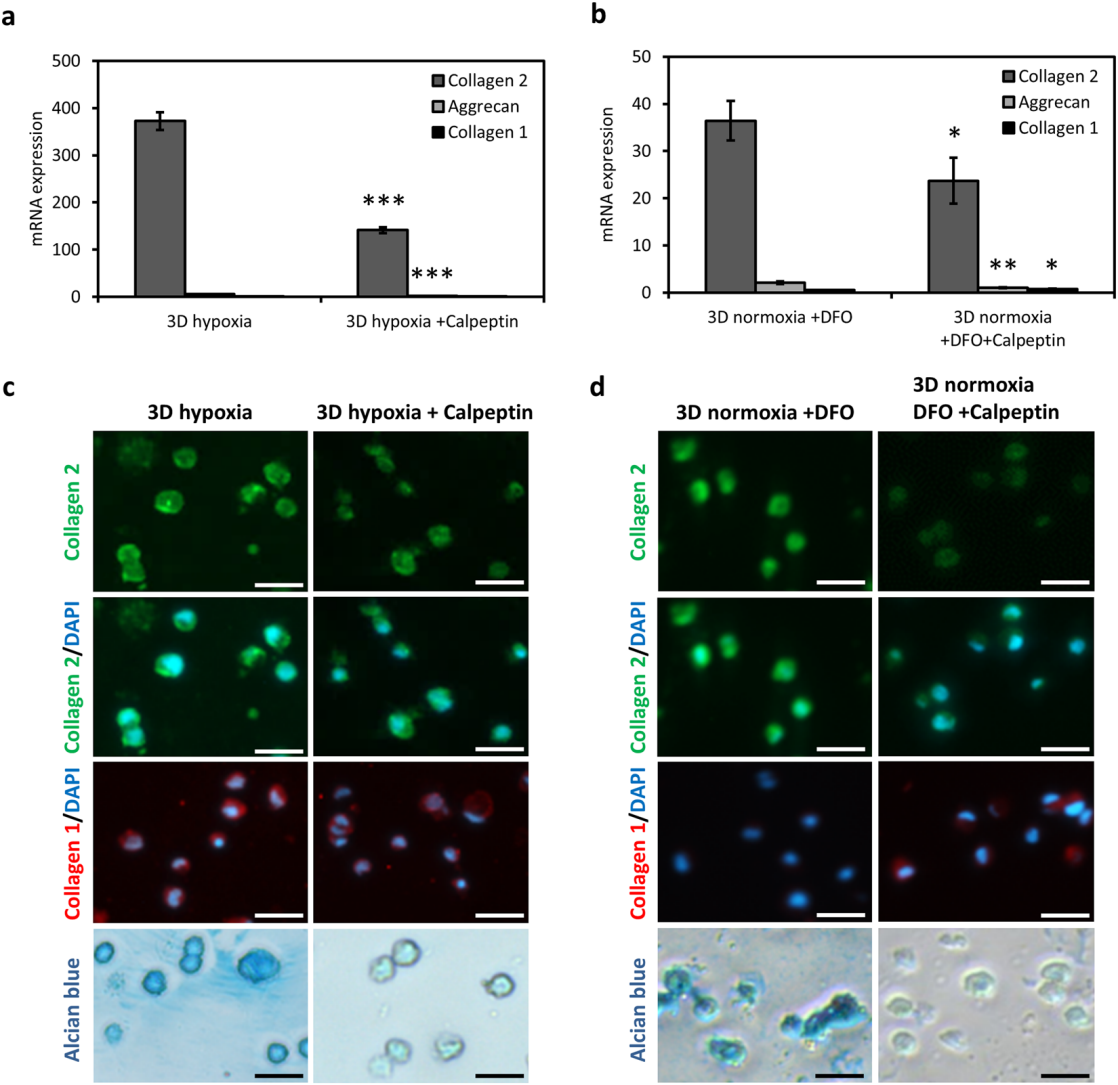
Calpeptin treatment diminishes hypoxia-induced re-differentiation of chondrocytes. (**a**) mRNA expression of collagen 2, collagen 1 and aggrecan of chondrocytes cultured in 3D under hypoxia with or without calpeptin (5 μM) treatment. Mean ± s.d. for n = 3; normalized to 3D normoxia control; ***p < 0.001 when compared to hypoxia. (**b**) mRNA expression of collagen 2, collagen 1 and aggrecan of chondrocytes cultured in 3D under normoxia with DFO (150 μM) or DFO and calpeptin (5 μM) treatment. Mean ± s.d. for n = 3; normalized to 3D normoxia control; *p < 0.05, **p < 0.01 when compared to 3D normoxia +DFO. (**c**) Immunofluorescence imaging of collagen 2 (green) and collagen 1 (red) and alcian blue staining of chondrocytes in 3D under hypoxia with or without calpeptin (5 μM) treatment. (**d**) Immunofluorescence imaging of collagen 2 (green) and collagen 1 (red) and alcian blue staining of chondrocytes in 3D under normoxia with DFO (150 μM) or DFO and calpeptin (5 μM) treatment. Scale bar: 25 µm.

We then investigated the contribution of hypoxia-induced suppression of RhoA in the re-differentiation of chondrocytes. We treated chondrocytes with a Rho activator, calpeptin, under hypoxic conditions in 3D and examined the expression of chondrogenic markers. Calpeptin treatment led to an upregulation of RhoA protein under both hypoxic and normoxic conditions in chondrocytes in 3D (Figure S4a). Furthermore, a strong decrease in the hypoxia-induced gene expression of collagen 2 and aggrecan was observed in response to calpeptin in 3D (Fig. 3a). Similarly, calpeptin treatment hindered the re-differentiation induced by HIF stabilization under normoxic conditions with DFO treatment (Fig. 3b). Moreover, calpeptin caused a slight but significant increase in collagen 1 gene expression in DFO-treated chondrocytes (Fig. 3b). Interestingly, on 2D, calpeptin treatment under hypoxia or under normoxia in the presence of DFO did not lead to significant changes in the expression of chondrogenic markers (Figure S4b). In line with the gene expression results, immunofluorescence and alcian blue stainings of chondrocytes in 3D showed that collagen 2 and GAG deposition was suppressed due to calpeptin treatment under both hypoxic conditions and under normoxia with DFO treatment (Fig. 3c,d). On the other hand, collagen 1 deposition seemed unaffected by calpeptin in chondrocytes in 3D and HIF-stable conditions (Fig. 3c,d). These results indicate that hypoxia or hypoxia-mimicking with DFO induce re-differentiation of chondrocytes in 3D at least partly via suppression of Rho in a HIF-dependent manner.

### Hypoxia causes a HIF-dependent suppression of β-catenin in 3D whereas induces it in 2D-cultured chondrocytes

**Figure 4:**
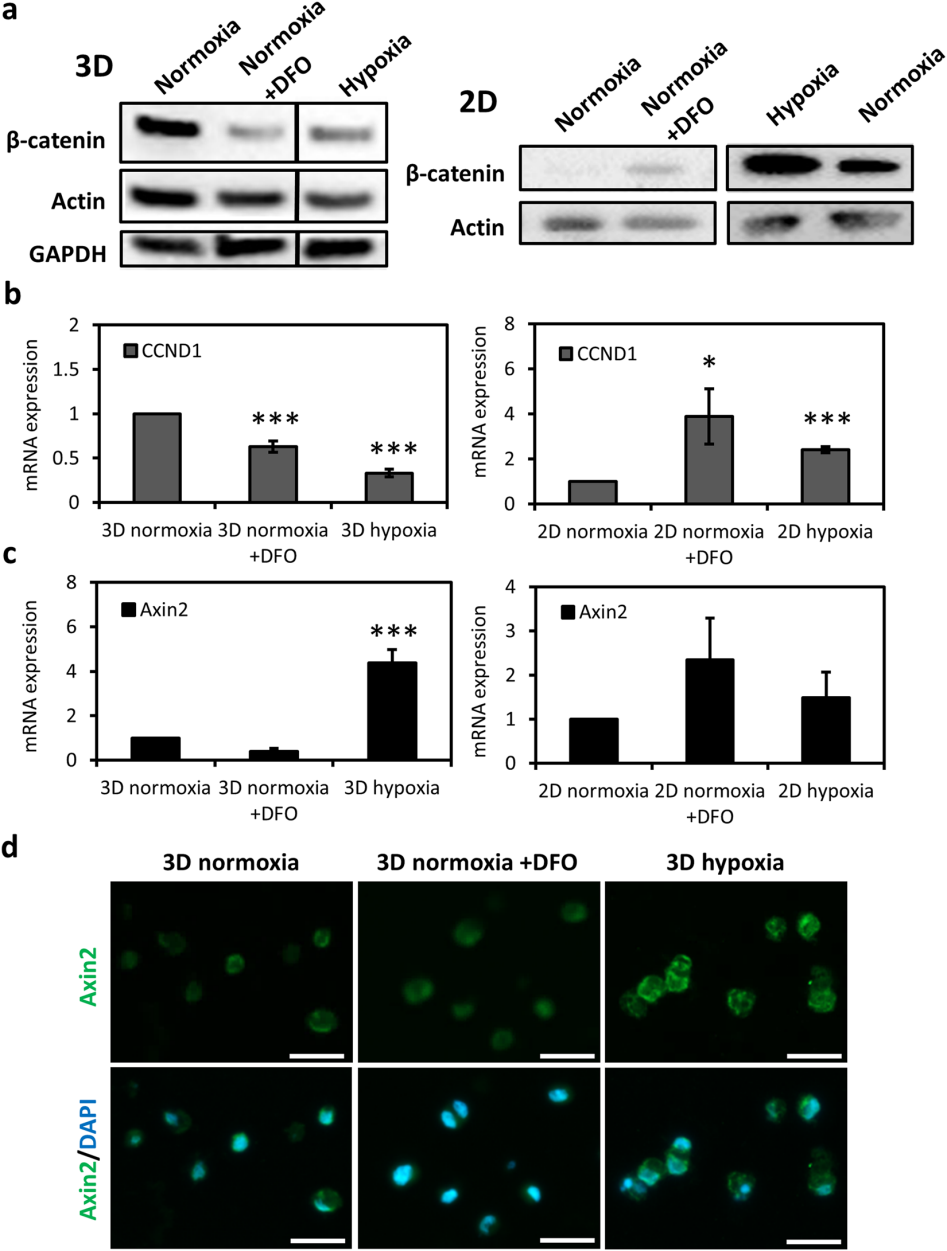
Hypoxia regulates β-catenin differently depending on the culturing conditions in a HIF-mediated manner. (**a**) Western blot showing β-catenin protein in chondrocytes in 3D and on 2D under hypoxia, normoxia and normoxia with DFO (150 µM) treatment. Actin and GAPDH was used as the loading control. (When extra lanes in between were removed in the blots, it is indicated with black line separation. Full blots are given in the Supplementary Information.) (**b**) mRNA expression of CCND1 and (**c**) Axin2 in chondrocytes in 3D or on 2D under hypoxia, normoxia and normoxia with DFO (150 µM) treatment. Mean ± s.d. for n = 3; normalized to normoxia; *p < 0.05, ***p < 0.001 when compared to normoxia. (**d**) Immunofluorescence imaging of Axin2 (green) in chondrocytes in 3D under hypoxia, normoxia and normoxia with DFO (150 µM) treatment. Scale bar: 25 µm.

We next explored the effect of hypoxia on Wnt/β-catenin signaling in chondrocytes under different culturing conditions. Hypoxia induced a potent suppression of β-catenin in chondrocytes in 3D hydrogels. Under normoxic conditions with DFO treatment, a similar loss of β-catenin protein levels was observed indicating that the effect was HIF-mediated (Fig. 4a). Interestingly, when the chondrocytes were cultured on 2D, hypoxia caused a strong increase of β-catenin protein levels in chondrocytes. DFO treatment under normoxia, on the other hand, caused a slight upregulation of β-catenin on 2D (Fig. 4a). We then looked into the expression of the target genes of Wnt/β-catenin pathway under different oxygen and culturing conditions. In line with the β-catenin protein levels, hypoxia or normoxia in the presence of DFO caused a downregulation of the target gene cyclin D1 (CCND1) in chondrocytes in 3D hydrogels (Fig. 4b). Similarly, CCND1 gene expression was upregulated under hypoxia or under normoxia with DFO in chondrocytes cultured on 2D (Fig. 4b). Contrarily, another target gene, Axin2, was strongly upregulated under hypoxia in chondrocytes cultured in 3D hydrogels, however, no such upregulation was observed in response to DFO treatment under normoxia (Fig. 4c). Immunofluorescence for Axin2 protein demonstrated a consistent trend to the gene expression data (Fig. 4d). On the other hand, Axin2 gene expression in chondrocytes cultured on 2D did not show a significant difference with changing oxygen conditions or HIF stability (Fig. 4c).

### Induction of Wnt signaling impedes hypoxia-induced re-differentiation of chondrocytes

**Figure 5:**
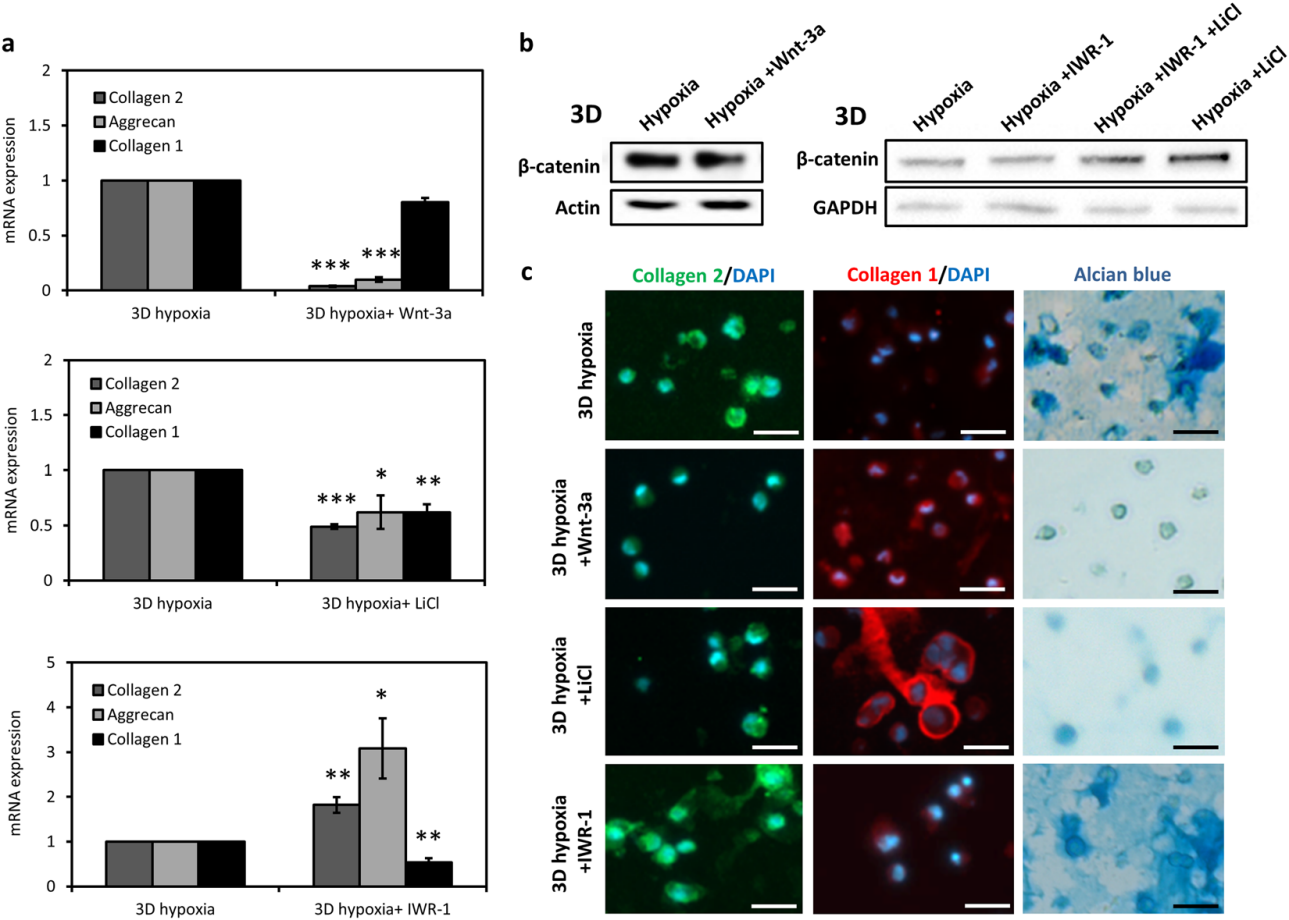
Induction of Wnt signaling suppresses whereas inhibition enhances hypoxia-induced re-differentiation of chondrocytes. (**a**) mRNA expression of collagen 2, collagen 1 and aggrecan of chondrocytes cultured in 3D under hypoxia with Wnt3a, LiCl (50 µM) and IWR-1 (5 µM) treatments. Mean ± s.d. for n = 3; normalized to 3D hypoxia control; *p < 0.05, **p < 0.01, ***p < 0.001 when compared to hypoxia control. (**b**) Western blots showing β-catenin protein in chondrocytes in 3D under hypoxia with Wnt3a, LiCl and IWR-1 treatments. Actin was used as the loading control. (**c**) Immunofluorescence imaging of collagen 2 (green) and collagen 1 (red) and alcian blue staining of chondrocytes in 3D under hypoxia with Wnt3a, LiCl and IWR-1 treatments. Scale bar: 25 µm.

In order to assess the involvement of β-catenin suppression in induction of chondrogenic re-differentiation under hypoxia in 3D, we next investigated the effect of manipulation of Wnt signaling on the expression of cartilage markers. First, we cultured chondrocytes in Wnt3a-conditioned medium under hypoxic conditions in 3D hydrogels. Upstream induction of Wnt signaling and β-catenin abrogated the hypoxia-mediated re-differentiation with a loss of collagen 2 and aggrecan gene expression and no effect on collagen 1 expression (Fig. 5a,b). Collagen 2 and GAG deposition were also suppressed with Wnt3a treatment (Fig. 5c). Then, we studied the effect of inducing canonical Wnt signaling in chondrocytes via inhibition of the β-catenin destruction complex with LiCl treatment. LiCl caused a strong increase in β-catenin protein levels in chondrocytes under hypoxic conditions in 3D and a significant decrease in the gene expression of cartilage markers collagen 2 and aggrecan (Fig. 5a,b). Hypoxia-induced deposition of collagen 2 and GAGs were also lost in response to LiCl (Fig. 5c). LiCl treatment also caused an increase in β-catenin protein in chondrocytes under normoxia in the presence of DFO (Figure S5a). Similarly, induction of β-catenin with LiCl dampened the DFO-mediated chondrogenic re-differentiation of chondrocytes in 3D (Figure S5b,c). As we found that hypoxia caused a strong increase in Axin2 expression, we treated chondrocytes with a stabilizer of Axin2 to investigate its effect on re-differentiation. IWR-1 has been identified as an inhibitor of canonical Wnt signaling through stabilizing Axin2 and leading to degradation of β-catenin^30^. IWR-1 treatment of chondrocytes caused a further increase in the gene expression of collagen 2 and aggrecan under hypoxic conditions whereas collagen 1 expression demonstrated a significant decrease (Fig. 5a). However, β-catenin protein levels were unaffected in chondrocytes in response to IWR-1 treatment under hypoxia (Fig. 5b). Consistent with the gene expression, IWR-1 treatment reinforced the hypoxia-induced collagen 2 and GAG deposition in chondrocytes (Fig. 5c). These results demonstrate that induction of Wnt signaling suppresses while inhibition of canonical Wnt signaling enhances the hypoxia-mediated re-differentiation of chondrocytes in 3D. Therefore, hypoxia-induced suppression of β-catenin within 3D microenvironment contributes to the regulation of chondrocyte phenotype.

## Discussion

Autologous cell-based cartilage therapies are challenged by chondrocyte de-differentiation due to monolayer expansion which leads to hindered reconstruction of cartilage-specific matrix. Understanding the signaling mechanisms that regulate de- and re-differentiation of chondrocytes is crucial to improve the outcome of current therapeutic approaches. Hypoxia is a strong inducer of chondrocyte phenotype through stabilization of HIFs, however, the hypoxia-induced down-stream signaling pathways that regulate the expression of chondrogenic markers are largely unknown. In this work, we demonstrate the hypoxic regulation of Rho and canonical Wnt pathways in a context-dependent manner in chondrocytes and their role in the maintenance of chondrogenic phenotype.

Our data shows that hypoxia induces chondrocyte re-differentiation in synergy with 3D culturing in line with previous reports^11, 31^. Hypoxic induction of chondrogenic markers is HIF-dependent as hypoxia mimicking via stabilization of HIF under normoxic conditions leads to a similar induction of re-differentiation, whereas destabilizing HIF under hypoxia abrogates the expression of chondrogenic markers. HIF-1α has been shown to be a crucial factor in cartilage development that regulates chondrocyte proliferation and cartilage matrix formation^20, 32^ as well as during re-differentiation of chondrocytes^23^. Similarly, HIF-2α has also been reported to mediate hypoxic re-differentiation via inducing Sox-9-driven expression of chondrocyte markers^24^. However, we found that hypoxia-induced stabilization of HIFs leads to potent expression of collagen 2 and aggrecan and suppresses collagen 1 only in 3D whereas on 2D, chondrogenic markers are mainly unaffected and collagen 1 is significantly upregulated. Cellular microenvironment and mechanosensing have been shown to be important factors for signaling of chondrocytes^33^. 3D culturing induces cell rounding, loss of actin stress fibers and downregulation of RhoA in chondrocytes and this loss of RhoA has been found to induce Sox-9-mediated re-differentiation in a PKA-dependent manner^28^. Moreover, RhoA overexpression has been shown to inhibit chondrogenic differentiation of mesenchymal cells^34, 35^. Therefore, we hypothesized a possible involvement of RhoA in hypoxia-induced re-differentiation of chondrocytes. We investigated the effect of hypoxia on the expression of RhoA in chondrocytes in 3D and on 2D. In 3D, chondrocytes suppress RhoA expression on both gene and protein levels in response to hypoxia in a HIF-mediated manner whereas on 2D hypoxia promotes RhoA. Hypoxia-induced effects on RhoA have only been addressed in a few studies in literature and they exclusively reported a positive regulation of RhoA. Hypoxia-triggered activation of RhoA signaling was shown to induce focal adhesions and motility in breast cancer cells^36^. Another study demonstrated upregulation of RhoA under low oxygen conditions in cardiac myocytes leading to increased calcium channels and ischemic arrhythmias^37^. Similar induction of RhoA by hypoxia was shown to be involved in cell spreading^38^, migration of mesenchymal cells^39^, protection of neuroblastoma cells from apoptosis^40^ and morphogenic branching of lung explants^41^. Here, we show for the first time that hypoxia can both induce and suppress RhoA signaling in chondrocytes depending on the cellular microenvironment. Furthermore, we found that the hypoxic inhibition of RhoA is involved in the re-expression of chondrogenic markers in 3D under hypoxia.

RhoA signaling has been shown to have a crosstalk with Wnt signaling to regulate a variety of cellular processes such as cell motility and differentiation^42, 43^. Induction of Wnt/β-catenin signaling via activation of RhoA has been reported to induce osteogenic differentiation of mesenchymal cells with increased transcriptional activity of β-catenin^43^. Furthermore, Wnt/β-catenin signaling is known to be a negative regulator of chondrocyte phenotype and chondrogenic differentiation through inhibition of Sox-9-mediated transctiption^29, 44, 45^. Hence, we also investigated the hypoxic regulation of Wnt/β-catenin pathway in chondrocytes. Similar to the regulation of RhoA, our data show that hypoxia has differential effects on the expression of β-catenin in chondrocytes. When the cells are cultured on 2D, hypoxia induces β-catenin whereas suppresses it in 3D in a HIF-dependent manner. Hypoxia has been shown to induce β-catenin activation and downstream signaling in stem cells^46^. Another study reported induction of Wnt11 in response to hypoxia to promote cellular proliferation and migration^47^. Moreover, β-catenin and HIF-1α interaction has been shown to regulate adaptation of tumor cells to hypoxia and drive HIF-mediated transcription^48^. On the other hand, HIF-1α-induced inhibition of Wnt signaling has also been reported in osteoblasts to control cell proliferation^49^. Interaction of HIF-1α and β-catenin was shown to suppress expression of matrix metalloprotease 13 (MMP13) and inhibit chondrocyte catabolism^50^. Our results show that hypoxia suppresses β-catenin signaling in chondrocytes in 3D which positively regulates expression of chondrogenic markers. Induction of canonical signaling in chondrocytes with Wnt-3a or LiCl treatment abrogates the hypoxia-induced re-differentiation of chondrocytes in 3D. Interestingly, when β-catenin is inhibited, Axin2, a known target of the β-catenin transcriptional activity, is potently expressed under hypoxic conditions in 3D whereas CCND1, another β-catenin target, is suppressed. Axin2 has been previously shown to regulate chondrocyte phenotype, enhance TGF-β signaling, promote collagen 2 expression and inhibit chondrocyte hypertrophy^51, 52^. Our data similarly demonstrate that an upregulation of Axin2 gene and protein expression under hypoxia accompanies chondrocyte re-differentiation and positively regulates chondrogenic phenotype in 3D. Furthermore, Axin2 stabilization with IWR-1 treatment exerts a synergistic effect with hypoxia on the expression of chondrogenic markers and promotes chondrocyte re-differentiation even on 2D (Fig. S6).

**Figure 6:**
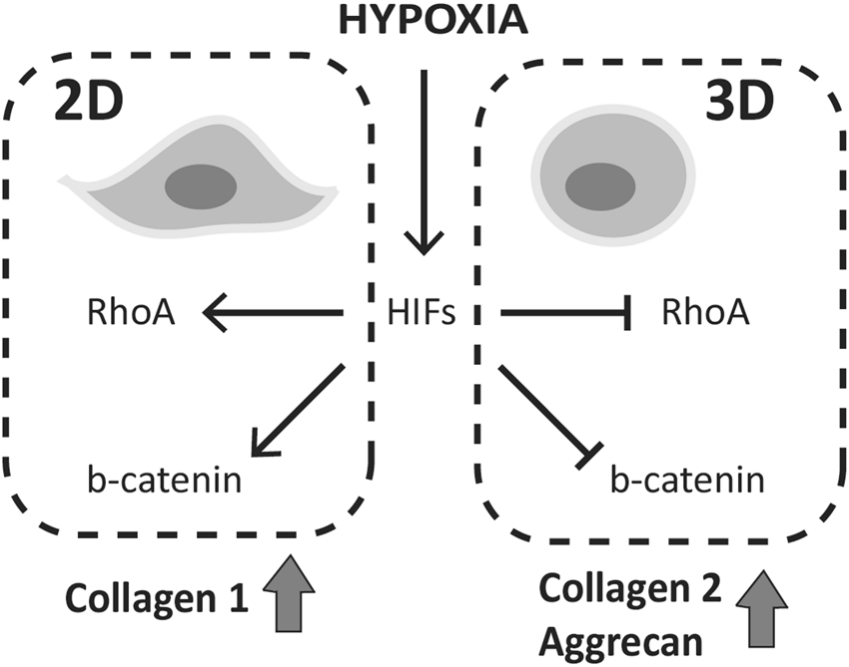
Scheme showing hypoxia-controlled expression of RhoA and β-catenin in chondrocytes. On 2D, hypoxia and HIF stabilization lead to an induction of RhoA and Wnt/β-catenin signaling in chondrocytes that leads to hindered re-differentiation and upregulation of collagen1. However, when chondrocytes are encapsulated in 3D hydrogels, RhoA and β-catenin are suppressed in response to hypoxia in a HIF-dependent manner with potent re-differentiation of chondrocytes marked by upregulation of collagen 2 and aggrecan.

In summary, this study shows the role of suppression of Rho and canonical Wnt signaling in the re-expression of chondrocyte phenotype under hypoxic conditions in 3D. These results contribute to the understanding of hypoxic regulation of signaling events in chondrocytes and development of new strategies for cell-based approaches in cartilage repair. Although we used severe hypoxia (1% O_2_) to mimic the oxygen tension in the deep zone chondrocytes, it would be of great interest to investigate whether similar effects would be observed within a range of hypoxic conditions. Moreover, this is the first demonstration of context-dependent regulation of RhoA and β-catenin in response to hypoxia and HIF stabilization (Fig. 6). Our results emphasize the importance of taking the cellular microenvironment and 3D context into consideration when studying regulation of signaling pathways under hypoxia. Therefore, the implications of this study are not limited to chondrocyte signaling but include other cellular phenomena where hypoxic signaling plays a crucial role such as stem cell differentiation and cancer.

## Materials and Methods

### Chondrocyte isolation and culture

Articular cartilage was removed from the knee joint of cows (1–2 years old, n = 3) with a sterile blade and minced. Cartilage pieces were washed with DMEM (Glutamax, high glucose) (Life Technologies) supplemented with 1% penicillin-streptomycin (P/S) (Gibco) and digested with 0.1% collagenase (Sigma) in DMEM supplemented with 5% fetal bovine serum (FBS) (Life Technologies) for 5 h at 37 °C with gentle shaking. Digested tissue was filtered with a 100 µm and then a 40 µm cell strainer followed by centrifugation at 500 g for 10 min and washed twice with culture medium (DMEM supplemented with 10% FBS and 50 µg/mL L-Ascorbic acid -2-phosphate (Sigma)). Chondrocytes were seeded in culture medium at a density of 5,000 cells/cm^2^ and medium was changed every 2–3 days. Cells were passaged at 80–90% confluence up to passage three.

### Re-differentiation of chondrocytes

At passage 3, the chondrocytes were either seeded onto tissue culture plastic (2D) or encapsulated into alginate hydrogels (3D). For 2D cultures, chondrocytes were seeded in culture medium at a density of 5,000 cells/cm^2^. For 3D encapsulation, chondrocyte suspension was mixed with sterile-filtered alginate (Novamatrix) solution in 150 mM NaCl at a final polymer concentration of 1% (w/v) and cell density of 6 × 10^6^ cells/mL. 30 µl discs of the mixture were pipetted onto gel casters and gelled in a 100 mM CaCl_2_ bath for 10 min. Chondrocytes on 2D or in alginate gels were cultured for in control medium or culture medium with the indicated drug treatments and cultured for 7 days under normoxic (21% O_2_) or hypoxic (1% O_2_) conditions.

### Drugs treatments used for the re-differentiation experiments

Chondrocyte culture medium was supplemented with the following drugs at the indicated concentrations: deferoxamine (DFO) (150 μM), YC-1 (10 μM), calpeptin (5 μM), lithium chloride (50 μM), IWR-1 (5 μM). All the drugs were purchased from Sigma. Wnt-3a-conditioned medium was produced as previously reported^53^ and used by mixing 1:1 with chondrocyte culture medium.

### Western blotting

For 2D samples, RIPA buffer with protease inhibitors (Sigma) was pipetted onto the cell monolayer, incubated on ice for 15 min and the lysate was removed with a scraper. For 3D samples, alginate gels were washed twice with 150 mM NaCl/5 mM CaCl_2_ and stored at −80 °C. Then, the frozen hydrogels were homogenized with an electrical pestle in RIPA buffer with protease inhibitors and incubated on ice for 30 minutes. Both 2D and 3D samples were then centrifuged at 10′000 g for 15 min, the supernatant was collected and the protein concentration was determined by Bradford assay (Bio-Rad). The samples were diluted with Laemmli buffer and RIPA and were kept at 95 °C for 5 min. 10–15 µg protein was loaded in pre-casted 4–12% Bis-Tris gels (Life Technologies) and run for 35 min at 125 V followed by transferring onto a nitrocellulose membrane for 1 h at 25 V. The membrane was washed twice with ddH_2_O, stained with Ponceau S (Sigma) for protein visualization and washed three times with tris-buffered saline with tween-20 (TBST) buffer. The membrane was blocked with 5% BSA for 1 h at RT and incubated with primary antibody overnight at 4 °C. The membrane was then washed four times with TBST for 8 minutes and incubated with the secondary antibody for 1 h at RT, washed again and visualized with Clarity Western ECL Substrate (Bio-Rad) for chemiluminescence. The primary antibodies used were anti-β-catenin (Abcam, ab32572; 1:1000), anti-RhoA (Santa Cruz, SC-418; 1:200), anti-actin (Sigma, A2066; 1:1000) and anti-GAPDH (Cell Signaling, 2118 S; 1:1000). The secondary antibodies used were anti-rabbit-HRP (Cell Signaling, 7074; 1:1000) and anti-mouse-HRP (Cell Signaling, 7076; 1:1000).

### Real-time quantitative PCR

For the 2D samples, lysis buffer (Macherey-Nagel AG) was added on the cells; the lysate was removed with a scraper and collected. For the 3D samples, alginate gels were collected, frozen and homogenized with an electrical pestle in lysis buffer. RNA was isolated with the NucleoSpin miRNA kit (Macherey-Nagel AG) according to the manufacturer’s instructions. RNA concentration was determined with a plate reader (Tek3 plate, Synergy, BioTek, Inc.). RNA was reverse transcribed and cDNA was amplified by quantitative real-time PCR (StepOnePlus, Applied Biosystems). Ribosomal protein L13 (RPL13a) was used as endogenous reference gene and fold change was quantified with the ΔΔCt method. The following primers for bovine (Microsynth AG) were used in this study: *RPL13a* (forward (F) 5′-GCCAAGATCCACTATCGGAAA-3′; reverse (R) 5′-AGGACCTCTGTGAATTTGCC-3′), *COL2A1* (Collagen 2) (F, 5′-GGCCAGCGTCCCCAAGAA-3′; R, 5′-AGCAGGCGCAGGAAGGTCAT-3′), *ACAN* (aggrecan) (F, 5′-GGGAGGAGACGACTGCAATC-3′; R, 5′-CCCATTCCGTCTTGTTTTCTG-3′), *COL1A2* (Collagen 1) (F, 5′-CGAGGGCAACAGCAGATTCACTTA-3′; R, 5′-GCAGGCGAGATGGCTTGTTTG-3′), *RHOA* (F, 5′-TGTATGTCCCTACGGTGTTTG-3′; R, 5′-CGATCATAATCTTCCTCGCCAG-3′), *CCND1 (cyclin D1)* (F, 5′-TTACACTGACAACTCCATCCG-3′; R, 5′-CATCTTGGAGAGGAAGTGCTC-3′), *Axin2* (F, 5′-GAACCTGAAGGATCGCAAAA-3′; R, 5′-GGTTTCAGCTGCTTGGAGAC-3′).

### Immunohistochemisty

For the 2D samples, the cells were washed with PBS and fixed with 4% formaldehyde with 0.1% Triton-X in PBS for 20 min at 4 °C. The samples were washed again twice with PBS and blocked 1 h with 5% BSA at RT followed by incubation with primary antibody overnight at 4 °C. Next, the samples were washed with PBS, incubated with secondary antibody for 1 h at RT, washed again with PBS, stained with DAPI and phalloidin-rhodamine for 15 min and imaged with fluorescence microscopy. For the 3D samples, alginate gels were fixed with 4% formaldehyde (Sigma) with 0.1% Triton-X (Sigma) in PBS for 1 h at 4 °C and washed twice with 150 mM NaCl/5 mM CaCl_2_. Then, the gels were kept in a 1:1 mixture of PBS and optimum cutting temperature compound (OCT, VWR) for 2 h at RT and then embedded in OCT overnight. The samples were frozen on dry ice and cut with a cryotome (CryoStar NX70, ThermoScientific) into 5 µm sections. The sections were washed with PBS and blocked with 5% BSA for 1 h at RT followed by incubation with primary antibody in 1% BSA overnight at 4 °C. Next, the sections were washed in PBS, incubated with the secondary antibody in 1% BSA for 1 h at RT, washed again and stained with DAPI for 15 min at RT. Then, the sections were mounted with aqueous mounting media (Vector Laboratories) and imaged with fluorescence microscopy. The primary antibodies used were anti-Collagen 2 (II-II6B3, DSHB, 1:20), anti-Collagen 1 (Abcam, ab6308, 1:200), anti-RhoA (Santa Cruz, SC-418; 1:200) and anti-Axin2 (Sigma, SAB3500619; 1:200). The secondary antibodies used were AlexaFluor 488 goat anti-mouse IgG and goat anti-rabbit IgG (Life Technologies, A10680, A11008, 1:300).

### Alcian blue staining

Both 2D samples and sections of 3D samples were washed with ddH_2_O and incubated with 3% acetic acid (Sigma) for 10 minutes. Then, the samples were incubated with alcian blue solution (Sigma) for 20 minutes, washed with ddH_2_O followed by imaging with light microscopy.

### Statistical analysis

Quantitative data are represented as mean ± standard deviation (s.d.) and obtained from three independent experiments. Analysis of variance (ANOVA) with Tukey’s and Bonferonni’s *post-hoc* tests were performed for statistical analyses and p values less than 0.05 were considered as statistically significant.

## Electronic supplementary material

Supplementary data
