# Supplementary material for: Hypoxia regulates RhoA and Wnt/β-catenin signaling in a context-dependent way to control re-differentiation of chondrocytes
Source: Sci Rep. 2017 Aug 22;7:9032. doi: 10.1038/s41598-017-09505-6 (PMC5567364; doi:10.1038/s41598-017-09505-6)
Supplement: Supplementary file 1 — Supplementary data [file 41598_2017_9505_MOESM1_ESM.pdf]

Hypoxia regulates RhoA and Wnt/β-catenin signaling in a context-dependent way to control re-differentiation of chondrocytes

Ece Öztürk[1], Stefanie Hobiger[1], Evelin Despot-Slade[1], Michael Pichler[1], and Marcy Zenobi-Wong[1,]*

[1] Cartilage Engineering+ Regeneration Laboratory, ETH Zurich, Otto-Stern-Weg 7, 8093, Zurich, Switzerland

*: Corresponding author (marcy.zenobi@hest.ethz.ch)

Supplementary Information

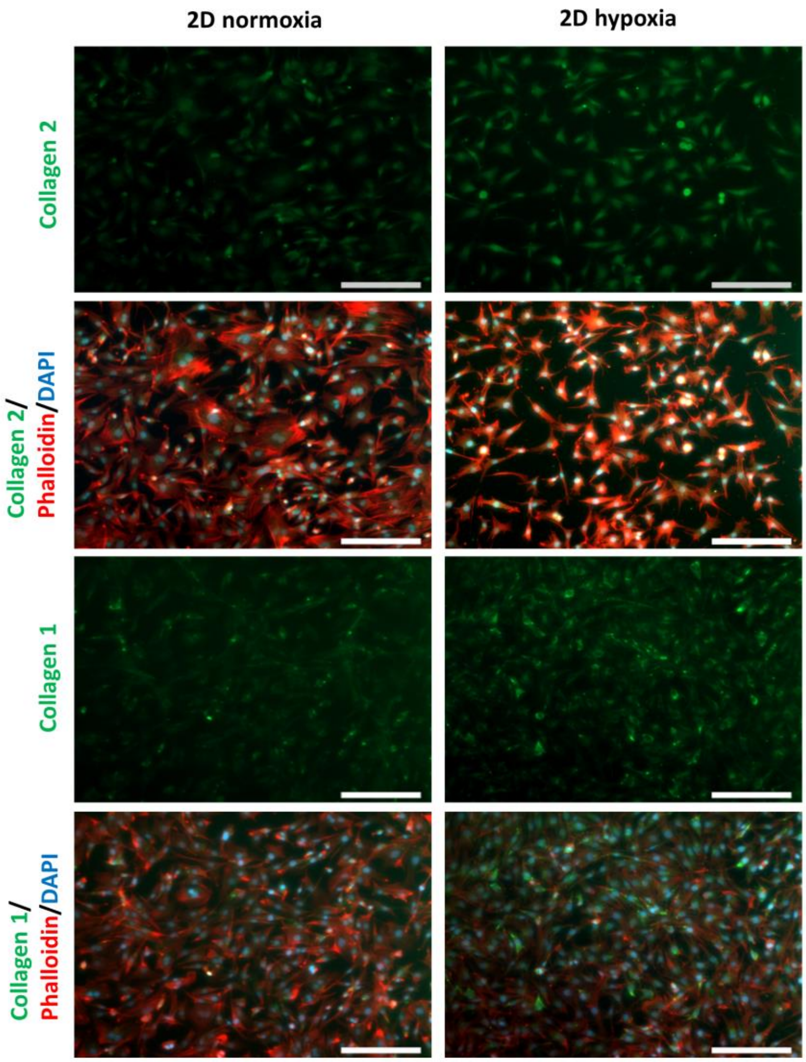

Figure S1: Effect of hypoxia on the expression of chondrogenic markers on 2D. Immunofluorescence imaging of collagen 2 (green) and collagen 1 (green) and alcian blue staining of chondrocytes on 2D under normoxia or hypoxia. DAPI (blue) was used for nuclei and phalloidin (red) was used for actin staining. Scale bar: 100 μm.

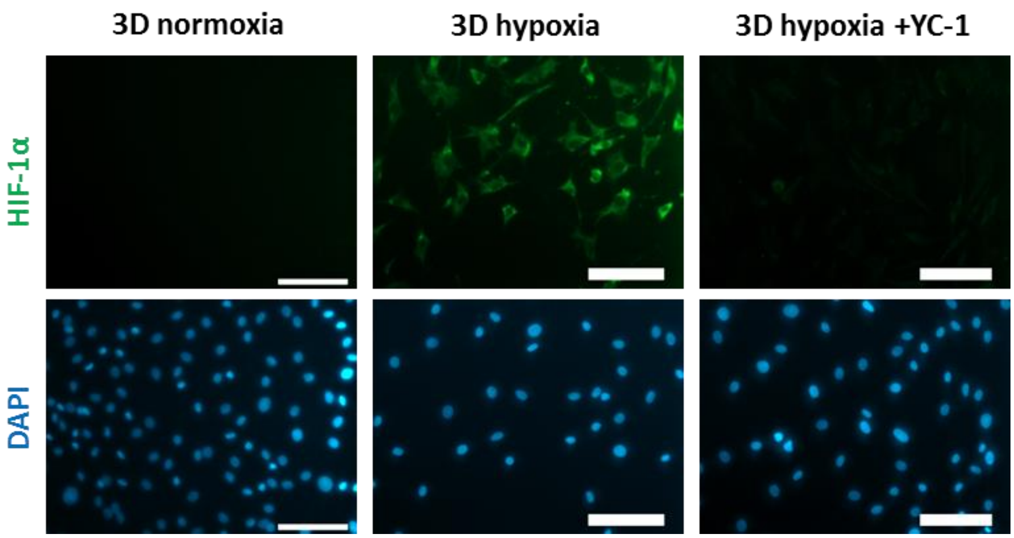

Figure S2: Stabilization of HIF-1α protein under hypoxic conditions in chondrocytes on 2D and destabilization of HIF-1α with YC-1 (10 µM) treatment. Scale bar: 50 µm.

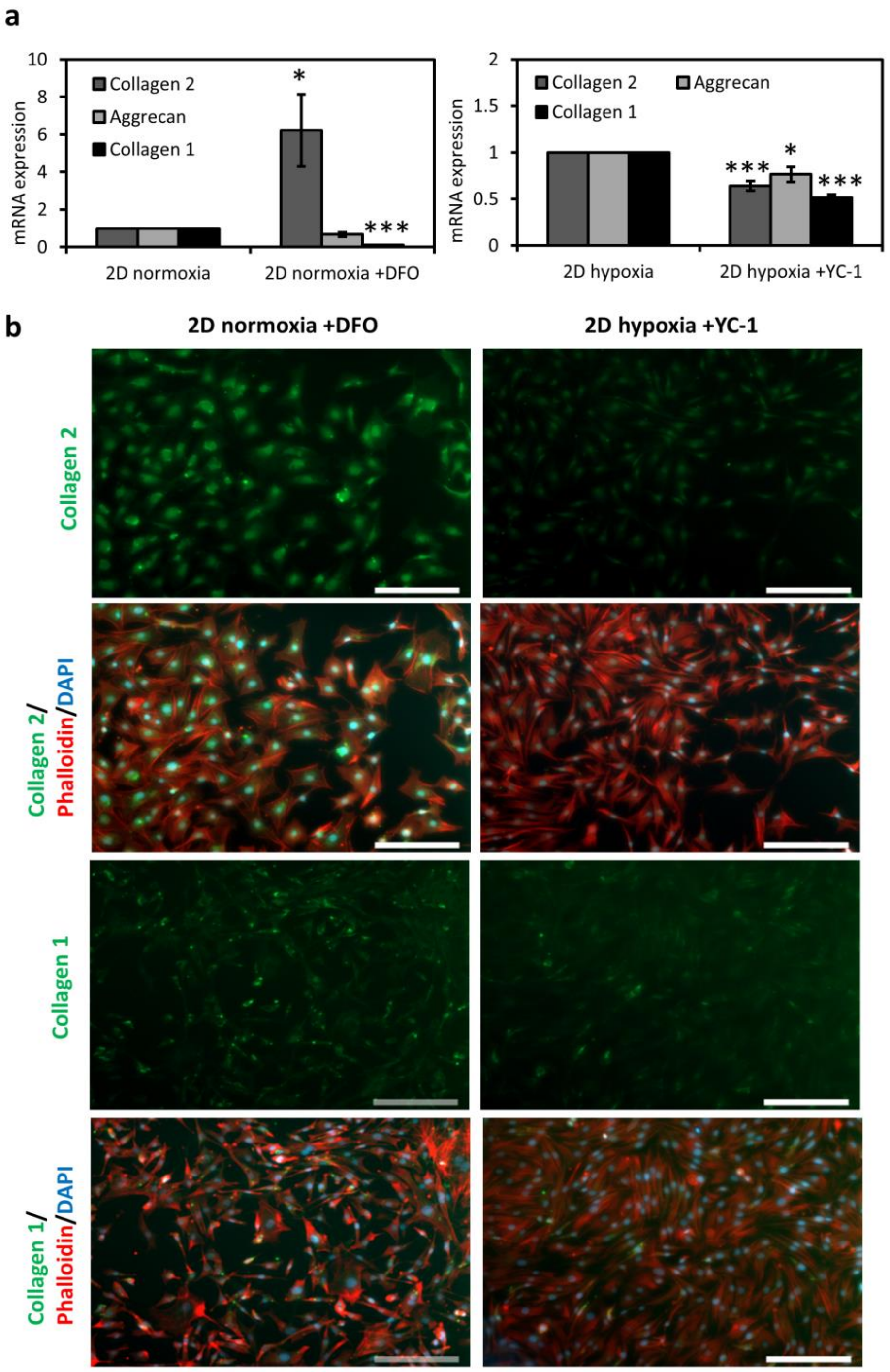

Figure S3: HIF-dependence of the expression of chondrogenic markers on 2D. (A) mRNA expression of collagen 2, collagen 1 and aggrecan of chondrocytes on 2D under normoxia with DFO (150 μM) treatment or under hypoxia with YC-1 (10 μM) treatment. *: p<0.05, ***: p<0.001 when compared to normoxia or hypoxia control. (B) Immunofluorescence imaging of collagen 2 (green) and collagen 1 (green) in chondrocytes on 2D under normoxia with DFO (150 μM) treatment or under hypoxia with YC-1 (10 μM) treatment. DAPI (blue) was used for nuclei and phalloidin (red) was used for actin staining. Scale bar: 50 μm.

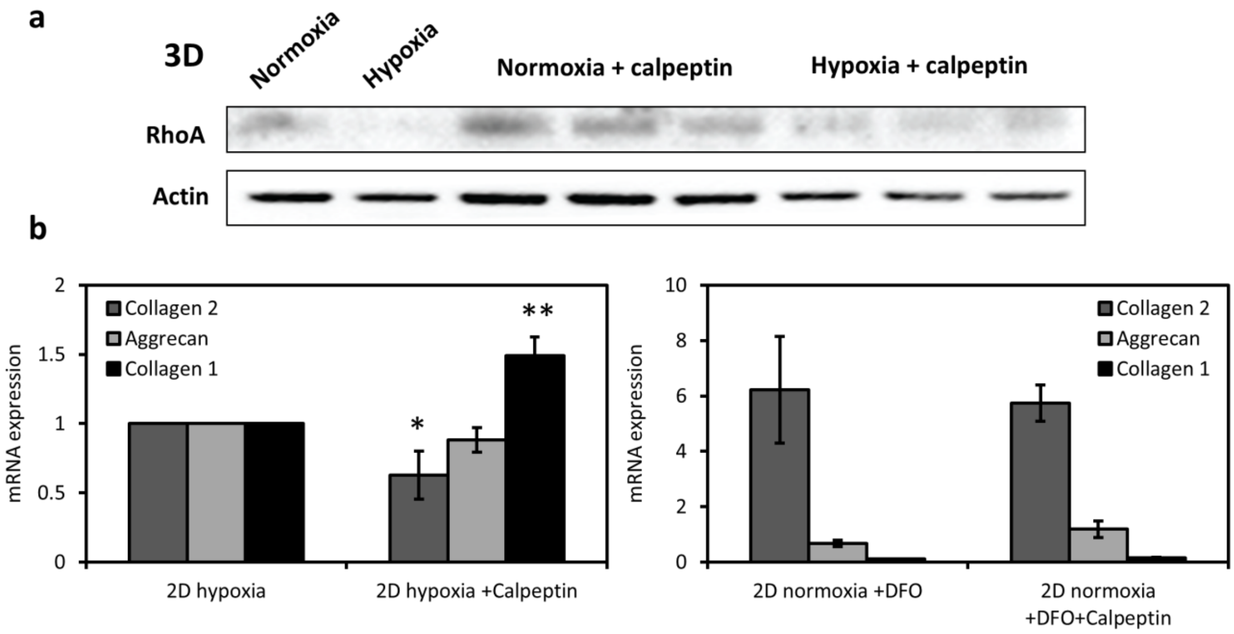

Figure S4: Effect of Rho activator calpeptin on chondrocytes. (A) Western blot showing total RhoA protein in chondrocytes in 3D under hypoxia and normoxia with or without calpeptin (5 µM) treatment. Actin was used as the loading control. (B) mRNA expression of collagen 2, collagen 1 and aggrecan of chondrocytes cultured on 2D under hypoxia with or without calpeptin (5 µM) or under normoxia with DFO (150 µM) or DFO and calpeptin (5 µM) treatment. *: $p<0.05$, **: $p<0.01$ when compared to hypoxia control.

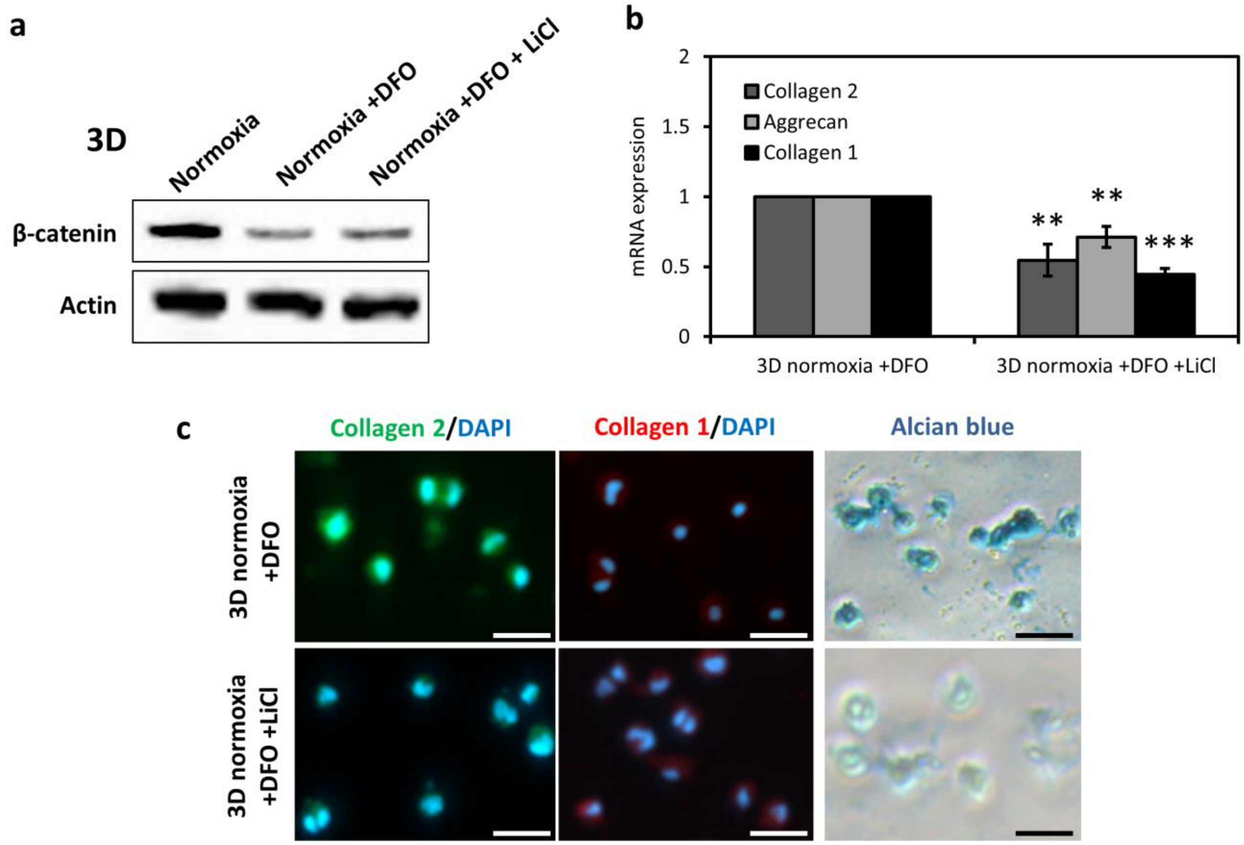

Figure S5: Effect of LiCl on the DFO-mediated suppression of β-catenin and the expression of chondrogenic markers in 3D. (A) Western blot of β-catenin protein in chondrocytes in 3D under normoxia, with DFO (150 µM) or DFO and LiCl (50 µM) treatment. Actin was used as the loading control. (B) mRNA expression of collagen 2, collagen 1 and aggrecan of chondrocytes cultured in 3D under normoxia with DFO (150 µM) or DFO and LiCl (50 µM) treatment. **: $p<0.01$, ***: $p<0.001$ when compared to normoxia with DFO. (C) Immunofluorescence imaging of collagen 2 (green) and collagen 1 (red) and alcian blue staining of chondrocytes in 3D under normoxia with DFO (150 µM) or DFO and LiCl (50 µM) treatment. DAPI (blue) was used for nuclei staining. Scale bar: 25 µm.

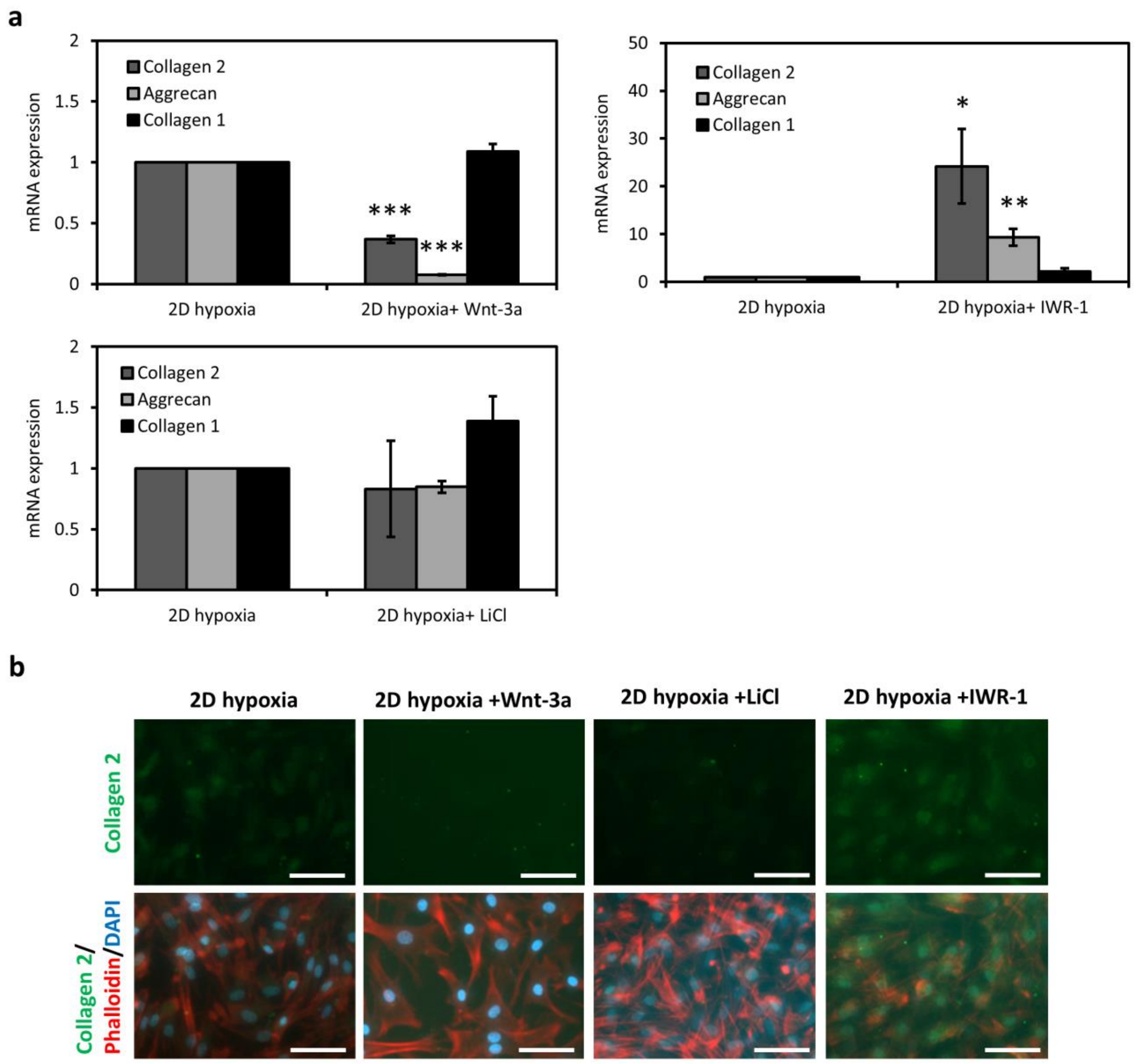

Figure S6: Manipulation of Wnt signaling in chondrocytes on 2D. (A) mRNA expression of collagen 2, collagen 1 and aggrecan of chondrocytes cultured on 2D under hypoxia with Wnt-3a, LiCl (50 μM) and IWR-1 (5 μM) treatments. *: p<0.05, **: p<0.01, ***: p<0.001 when compared to hypoxia control. (B) Immunofluorescence imaging of collagen 2 (green) in chondrocytes on 2D under hypoxia with Wnt-3a, LiCl and IWR-1 treatments. Scale bar: 25 μm.

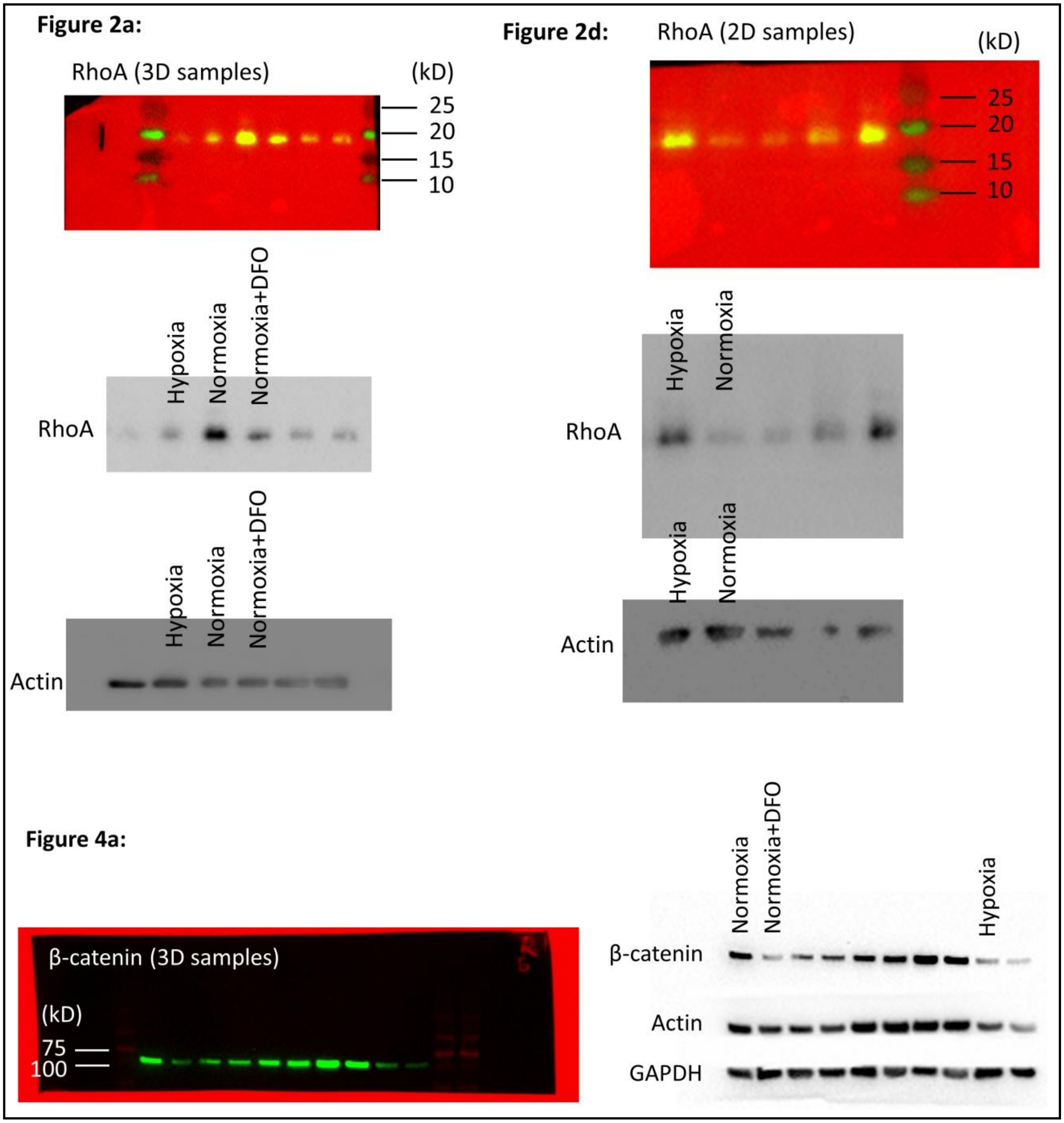

Figure S7: Selected Western blots from the main text. The blots in Figure 4A were cropped and merged for clarity indicating the Normoxia, Normoxia+DFO and Hypoxia samples.
